# Epithelial-Mesenchymal Transition Activates YAP to Drive Malignant Progression and Immune Evasion

**DOI:** 10.3390/cancers17172767

**Published:** 2025-08-25

**Authors:** Xi Huang, Mingyan Zhang, Alexander D. Pearce, Matthew D. Gibbons, Dan Jin, Lu Li, Dongxin Hu, Renbin Liu, Mu Yu, Ming Tan, Jia Chang, Jixin Dong, Mingyi Xie, Weizhou Zhang, Lizi Wu, Catherine Flores, Jörg Bungert, Todd M. Brusko, Jianrong Lu

**Affiliations:** 1 Department of Biochemistry and Molecular Biology, College of Medicine, University of Florida, Gainesville, FL 32610, USA; xi.huang@ufl.edu (X.H.); zhangmingyan@sdfmu.edu.cn (M.Z.); matthewgibbons@ufl.edu (M.D.G.); luli1@ufl.edu (L.L.); hudongxin@sdfmu.edu.cn (D.H.); renbinliu@ufl.edu (R.L.); mingyi.xie@ufl.edu (M.X.); jbungert@ufl.edu (J.B.); 2 Shandong Provincial Hospital Affiliated to Shandong First Medical University, Jinan 250021, China; 3 Department of Pathology, Immunology and Laboratory Medicine, College of Medicine, University of Florida, Gainesville, FL 32610, USA; apearce1@ufl.edu (A.D.P.); zhangw@ufl.edu (W.Z.); tbrusko@ufl.edu (T.M.B.); 4 Lillian S. Wells Department of Neurosurgery, College of Medicine, University of Florida, Gainesville, FL 32610, USA; dan.jin@neurosurgery.ufl.edu (D.J.); catherine.flores@neurosurgery.ufl.edu (C.F.); 5 Department of Molecular Genetics and Microbiology, College of Medicine, University of Florida, Gainesville, FL 32610, USA; yumu@ufl.edu (M.Y.); lzwu@ufl.edu (L.W.); 6 Institute of Biochemistry and Molecular Biology, Research Center for Cancer Biology, China Medical University, Taichung 406040, Taiwan; mingtan@cmu.edu.tw; 7 Section of Periodontics, UCLA School of Dentistry, Los Angeles, CA 90095, USA; jiachang@dentistry.ucla.edu; 8 Eppley Institute for Research in Cancer and Allied Diseases, Fred & Pamela Buffett Cancer Center, University of Nebraska Medical Center, Omaha, NE 68198, USA; dongj@unmc.edu

**Keywords:** EMT, hippo signaling, immune checkpoint

## Abstract

**Background**: Epithelial-mesenchymal transition (EMT) is prevalent in human cancer and facilitates tumor metastasis and therapy resistance by enhancing cancer cell motility, invasiveness, survival, and immune evasion. However, the molecular mechanisms underlying the cellular changes during EMT remain largely elusive, making it challenging to simultaneously target these diverse malignant phenotypes. **Results**: Here, we show that the EMT-inducing ZEB transcription factors directly repressed WWC1 (also known as KIBRA), a key upstream activating component of the Hippo signaling pathway. The EMT program thus inherently downregulated WWC1, leading to impaired Hippo signaling and constitutive activation of the downstream effector and transcriptional coactivator YAP. The YAP-dependent transcriptional program promotes manifold cellular phenotypes that resemble those induced during EMT. Indeed, pharmacological inhibition of YAP suppressed EMT-stimulated cell migration and invasion, apoptosis resistance, and cell size growth, identifying active YAP as a common essential mediator of multiple EMT-associated phenotypes. Moreover, YAP activation directly induced transcription of B7 family immune checkpoint proteins VSIR (VISTA) and PD-L2, and rendered cancer cells resistant to effector CD8 T cells. **Conclusions**: Collectively, the results suggest that EMT intrinsically activates YAP by repressing WWC1, providing a non-genetic mechanism for pervasive YAP activation in cancer. Activated YAP, in turn, critically contributes to diverse EMT-enhanced malignant phenotypes and immune evasion. Therefore, pharmacological targeting of YAP may suppress various EMT-associated malignant properties and improve the efficacy of anti-PD-1 immunotherapy, offering a promising therapeutic strategy against cancer cells exhibiting EMT characteristics.

## Simple Summary

Cancer cells acquire various enhanced malignant properties by undergoing epithelial-mesenchymal transition (EMT). However, the underlying mechanism remains largely unclear, and it is difficult to target the diverse malignant phenotypes. We found that EMT inherently repressed the expression of WWC1, a key upstream activating component in Hippo signaling, thus impeding this pathway and activating its downstream effector YAP. YAP activation was required for the acquisition of EMT-stimulated cell migration and invasion, resistance to apoptosis, and cell size growth. YAP also directly induced immune checkpoint proteins VISTA and PD-L2, and rendered cancer cells resistant to effector CD8 T cells. The results suggest that EMT activates YAP, which in turn critically mediates multiple EMT-associated malignant phenotypes and immune evasion.

## 1. Introduction

Most human cancers arise from epithelial tissues. Epithelial cells exhibit remarkable plasticity and, under proper stimulating cues, can undergo a reversible process known as epithelial-mesenchymal transition (EMT) [1]. Through EMT, epithelial cells shed their epithelial characteristics, such as cell polarity and cell-cell junctions, and acquire the properties of mesenchymal cells, such as enhanced motility, invasiveness, and resistance to apoptosis. Various pro-EMT signaling pathways (e.g., TGFβ) induce the expression of key EMT-driving transcription factors (EMT-TFs), mainly SNAI1/2, ZEB1/2, and TWIST, which repress epithelial genes and activate mesenchymal genes, resulting in a variable extent of EMT [1]. Single-cell gene expression analyses revealed that EMT is a nearly universal phenomenon in major types of human carcinomas [2]. EMT reprograms global gene expression and signaling networks, endows cancer cells with increased migratory and invasive capacities, survival, and immune suppression, thus facilitating metastatic spreading and therapy resistance [3]. Cancer cells undergoing a partial EMT critically contribute to metastasis formation [4,5,6]. EMT also promotes resistance to chemotherapy, targeted therapy, and immunotherapy [3,7]. Due to its prevalence and prominent role in cancer, EMT has emerged as a potential therapeutic target of prime interest. Two recent studies showed that the embryonic factor netrin-1 exhibited pro-EMT activities, and blocking netrin-1 with a monoclonal antibody inhibited EMT, increased the proportion of epithelial cells, reduced metastasis, and potentiated therapy efficacy in mouse models of skin squamous cell carcinoma and endometrial adenocarcinoma [8,9]. These studies validate the clinical significance of targeting EMT. However, there are still very few pharmacological approaches available to suppress EMT or destroy cancer cells that have undergone EMT. No therapeutic interventions that specifically target EMT are used in clinical oncology practice. The molecular mechanisms by which EMT induces diverse malignant properties remain inadequately understood. Moreover, EMT-stimulated malignant phenotypes are manifold, making it challenging to target cancer cells exhibiting EMT. Uncovering key regulatory mechanisms underlying EMT-associated phenotypes will help develop anti-EMT therapeutic strategies.

The evolutionarily conserved Hippo signal transduction pathway was originally characterized in the fruit fly. The core of the Hippo pathway in mammals consists of a kinase cascade in which the protein kinases MST1/2 (Hippo in fly) phosphorylate and activate the LATS1/2 kinases, which then phosphorylate the main downstream effectors—the YAP/TAZ transcriptional coactivators—causing their cytoplasmic retention and consequent inactivation [10]. Hippo signaling activity is tightly regulated by a wide range of signals [10,11]. When Hippo signaling (i.e., the kinase cascade) is inactive, YAP/TAZ are hypo-phosphorylated and localize in the nucleus where they interact with the TEAD transcription factors to activate their target genes. WWC1 (KIBRA) was identified as a key upstream activator component of the Hippo signaling pathway in flies in vivo [12,13,14]. Loss of WWC1 phenocopied loss of canonical Hippo signaling in *Drosophila*. Biochemically, WWC1 is a multi-domain scaffold protein [15] and acts as an organizer by interacting with LATS1/2, Merlin (NF2), SAV1, and multiple other components in the Hippo pathway to form supramolecular complexes/condensates, which facilitate phosphorylation and activation of LATS1/2 [12,13,14,16,17,18,19,20]. The Hippo pathway plays a pivotal role in regulating organ size, tissue growth, regeneration, and fibrosis. Although genetic alterations in the core components of the Hippo pathway are infrequent, dysregulation of Hippo signaling and hyperactivation of YAP/TAZ are pervasive in human cancer, which transcriptionally drive cell proliferation, migration, invasion, and survival, leading to tumor development, metastasis, and therapy resistance [10,21,22]. Such malignant phenotypes largely mimic those induced during EMT. In addition, activation of YAP/TAZ in cancer cells exerts complex effects on immune responses [23]. LATS1/2 deletion that activated YAP/TAZ in mouse cancer cells enhanced anti-tumor adaptive immunity and led to tumor destruction [24]. On the contrary, YAP activation in cancer cells upregulated multiple cytokines/chemokines to promote the recruitment and differentiation of myeloid-derived suppressor cells (MDSCs) [25,26,27]. Tumoral YAP/TAZ also induced the recruitment and polarization of immunosuppressive type II (M2) tumor-associated macrophages (TAMs) [28,29,30,31]. Moreover, YAP/TAZ activation in human cancer cells directly upregulated the expression of immune checkpoint PD-L1 (CD274, B7-H1) to inhibit T cell function and cause immune evasion [32,33,34,35,36].

In the present study, we uncovered that WWC1 was a direct target for transcriptional repression by EMT-TFs, and thus was inherently downregulated by EMT. Repression of WWC1 impaired LATS activation, leading to constitutive activation of YAP. Therefore, EMT may represent a non-genetic mechanism for YAP activation in carcinomas. Pharmacological blockade of TEAD-YAP abrogated multiple EMT-enhanced malignant phenotypes without morphologically reversing EMT. The results suggest that EMT activates YAP, and active YAP in turn mediates diverse EMT-associated phenotypes. YAP activation also induced B7 family immune checkpoints VSIR (VISTA, B7-H5) and PD-L2 (CD273, B7-DC) [37], conferring resistance to effector T cells. Overall, the study suggests that EMT promotes new malignant properties and immune evasion by activating YAP, and highlights YAP as an attractive target for therapeutic intervention of cancer cells exhibiting EMT.

## 2. Materials and Methods

Cell Culture and Treatment. AsPC1, breast cells (BT474, Hs578T, MCF7, MDA-MB-231, NMuMG), and lung cancer cells (A549, H1437, H1573, H1819) (from ATCC) were cultured in Dulbecco’s Modified Eagle Medium (DMEM) supplemented with 10% fetal bovine serum (FBS) and penicillin-streptomycin. DCIS-Snai1-ER cells were previously established and were maintained in DMEM/F12 supplemented with 5% horse serum. Cells were treated with recombinant human TGFβ1 protein (R&D SYSTEMS, Minneapolis, MN, USA, 7754-BH), 4-hydroxytamoxifen (Sigma, St. Louis, MO, USA, H6278), verteporfin (TargetMol, Boston, MA, USA, T3112), MGH-CP1 (TargetMol, T9032), CA3 (Sigma, SML2647), VT104 (TargetMol, T67872), JQ-1 (TargetMol, T2110), ABT-737 (Apexbio, Boston, MA, USA, A8193), TRULI (Cayman Chemical, Ann Arbor, MI, USA, 36623), and TDI-011536 (TargetMol, T60144).

Antibodies. Immunofluorescence: YAP (D8H1X) XP Rabbit mAb (Cell Signaling, Danvers, MA, USA, 14074). Goat anti-Rabbit IgG (H+L) Highly Cross-Adsorbed Secondary Antibody, Alexa Fluor™ Plus 488 (Invitrogen, Waltham, MA, USA, A32731). Western blotting: Phospho-YAP (S127) Antibody (Cell Signaling, 4911), VSIR/VISTA antibody (Cell Signaling, 64953), E7 anti-β tubulin (DSHB, Iowa City, IA, USA), Mouse Anti-E-Cadherin (BD Biosciences, San Jose, CA, USA, 610181), Human-Serum-Adsorbed and Peroxidase-Labeled Anti-Mouse IgG (H+L) Antibody (LGC, Milford, MA, USA, 074-1806), and Human-Serum-Adsorbed and Peroxidase-Labeled Anti-Rabbit IgG (H+L) Antibody (LGC, 074-1516).

RNA isolation, reverse transcription, and real-time PCR. Total RNA was isolated using TRIzol reagent (Invitrogen) according to the manufacturer’s protocol. RNA concentration and purity were measured by nano-drop. cDNA was synthesized from 1 µg of total RNA using Moloney murine leukemia virus (M-MuLV) reverse transcriptase (NEB, Ipswich, MA, USA, M0253). Real-time PCR was then performed with PowerUp SYBR Green master mix (Applied Biosystems, Foster City, CA, USA, A25741). Relative mRNA expression was calculated using the comparative ΔΔCt method and normalized to β-actin levels.

Immunofluorescence. Cells were seeded on coverslips and treated with TGFβ or mock-treated for 2 days, followed by serum starvation for 1 day. Cells were washed with PBS, fixed with 4% formaldehyde, and permeabilized with 0.25% Triton X-100. Coverslips were then incubated with anti-YAP antibody overnight at 4 °C, followed by secondary antibody incubation for 1 h at 37 °C. Coverslips were mounted on slides and visualized under a fluorescence microscope.

Western blotting. Cells were lysed in lysis buffer containing 2% SDS and boiled for 20 min. Cell lysates were separated on 10% SDS-PAGE gels before being transferred onto Immobilon-P PVDF Membrane (Millipore, Burlington, MA, USA, IPVH00010). Membranes were blocked with 5% non-fat milk for 1 h and incubated with primary antibodies overnight at 4 °C. Following washes with 1× TBST, membranes were incubated with secondary antibodies for 1 h. Signals were detected using SuperSignal™ West Pico PLUS Chemiluminescent Substrate (Thermo Scientific, Waltham, MA, USA, 34580).

ChIP assay. At least 5 × 10^6^ cells were collected and crosslinked with 1% formaldehyde at room temperature for 10 min, followed by quenching with 125 mM glycine. Cells were then resuspended in cell lysis buffer and nuclei lysis buffer. Chromatin was sheared to 200–500 bp fragments on ice using a Bioruptor disruptor (Diagenode, Denville, NJ, USA). Sheared chromatin was incubated overnight with Flag antibody (Sigma, F1804) and protein A/G magnetic beads (Medchemexpress, Monmouth Junction, NJ, USA, HY-K0202) rotating at 4 °C. After extensive washing, crosslinks were reversed, and DNA was purified using a PCR purification kit and subjected to real-time PCR as described above.

Transwell migration and invasion assay. Materials: Matrigel Growth Factor Reduced Basement Membrane Matrix (Corning, Corning, NY, USA, 356231) and 6.5 mm Transwell with 8.0 µm Pore Polycarbonate Membrane Insert (Costar, Kennebunk, ME, USA, 3422). Cells were treated with or without TGFβ/4-HT for 2 days, resuspended in serum-free media, and seeded into Transwell inserts containing TGFβ/4HT and YAP-TEAD inhibitors. Inserts were placed into lower chambers containing media supplemented with 10% FBS and incubated for 24 h. Non-migrated cells were removed with cotton swabs, while migrated cells were fixed with 4% formaldehyde in PBS and stained with Crystal Violet. For the invasion assay, chambers were pre-coated with Matrigel matrix before cell seeding.

Flow cytometry. At least 1 × 10^6^ cells per group were collected and resuspended in pre-cold PBS. Cell size was determined by forward scatter using the BD Accuri C6 Plus Flow Cytometer (BD Biosciences).

Human T cell cytotoxicity assay. Naïve CD8^+^ T cells were isolated from leukapheresis-derived blood of healthy donors obtained from LifeSouth Community Blood Centers (Gainesville, FL, USA), using the EasySep Human Naïve CD8^+^ T Cell Isolation Kit (Stemcell, Cambridge, MA, USA, 19258). The isolated cells were activated with Human T-Activator CD3/CD28 Dynabeads (Thermo Fisher, 11161D) at a 1:1 bead-to-cell ratio in the presence of 100 IU/mL IL-2 and 5 ng/mL IL-7 (R&D Systems). After 48 h of incubation, beads and cytokines were removed. Activated CD8^+^ T cells were co-cultured with pre-seeded tumor cells at effector-to-target (E:T) ratios of 1:1, 5:1, and 10:1. Following a 24-h incubation, non-adherent T cells were removed by washing with PBS. Remaining adherent tumor cells were stained with 2μM Calcein-AM (AAT Bioquest, Pleasanton, CA, USA, 22003) and quantified to assess viability.

## 3. Results

### 3.1. WWC1 Is a Direct Transcriptional Target of ZEB Transcription Factors and Is Downregulated During EMT

**Figure 1:**
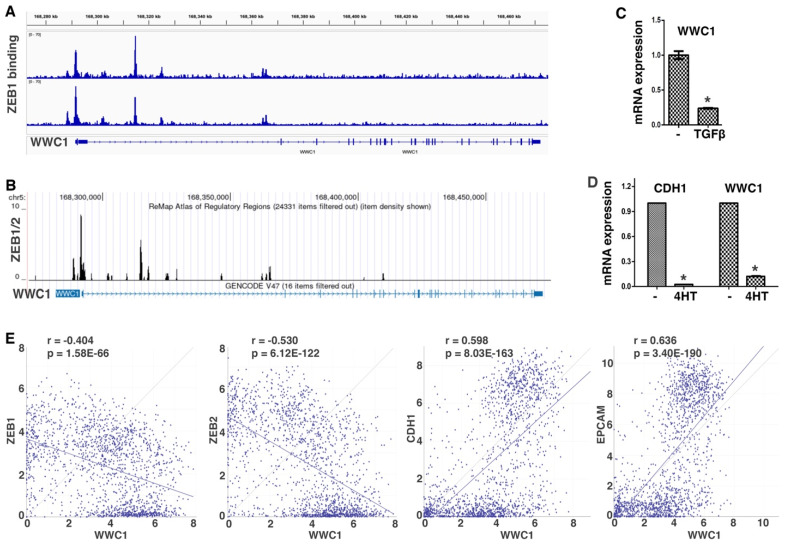
WWC1 is a direct transcriptional target of EMT-TF ZEB1. (**A**) ZEB1 genomic binding at *WWC1* in MDA-MB-231 human breast cancer cells in replicate (based on ZEB1 ChIP-seq data at SRA: ERP116911, or ArrayExpress: E-MTAB-8258). (**B**) ReMap ChIP-seq analysis of ZEB1/2 at the *WWC1* locus (UCSC genome browser). (**C**) WWC1 expression in NMuMG mouse mammary epithelial cells following TGFβ treatment. NMuMG cells were treated with TGFβ (5 ng/mL) or mock-treated for 2 days, then subjected to quantitative RT-PCR analysis (normalized against β-actin). Error bars represent S.D. * *p* < 0.05. (**D**) Expression of WWC1 and CDH1 in MCF10DCIS.com human breast cancer cells following Snai1-induced EMT. DCIS-Sna-ER cells (MCF10DCIS.com cells stably expressing inducible Snai1-ER) were cultured in the presence of 4-hydroxytamoxifen (4HT, 100 nM) or vehicle (DMSO) for over 2 weeks. Gene expression was measured by RT-PCR (normalized against β-actin). (**E**) Correlations between expression of WWC1 and EMT markers. Plots generated by DepMap Portal show expression correlations between WWC1 and indicated epithelial/mesenchymal markers in 1673 human cell lines. Linear regression lines, Pearson correlation (r), and *p*-value of linear regression are shown. Each dot represents a cell line. The x-axis shows WWC1 expression (log2), and the y-axis shows expression (log2) of the indicated markers.

ZEB and SNAIL families of EMT-TFs directly repress a large number of epithelial-enriched genes for EMT induction. To characterize EMT-associated phenotypes, we sought to identify new important genomic targets of EMT-TFs. We analyzed ChIP-seq data of ZEB1 genomic binding in mesenchymal triple-negative breast cancer (TNBC) cell line MDA-MB-231 [38]. Robust binding of ZEB1 was observed at the proximal promoter and intron 1 of WWC1 (Figure 1A), which encodes a key upstream activating component of the Hippo pathway. A specific E-box motif (5′-CACCTG), the consensus binding sequence for ZEB, was present at the center of both ZEB1 binding peaks, suggesting that the binding is direct. The ReMap database consists of a large-scale integrative analysis of all public ChIP-seq data for various transcriptional regulators in a wide variety of cell types [39]. Based on the ReMap data, ZEB1/2 displayed binding to the WWC1 locus in multiple human cell lines (Figure 1B). Weaker binding of SNAI2 at this genomic region (mostly in intron 1 of WWC1) was also detected. The data suggest that WWC1 is a direct genomic target of EMT-TFs ZEB and SNAIL.

ZEB and SNAIL generally act as transcriptional repressors and repress the expression of many epithelial genes during EMT. Given their binding at the *WWC1* locus, we determined if WWC1 expression was regulated by EMT. TGFβ is a potent EMT inducer and can upregulate multiple EMT-TFs [1]. TGFβ induces particularly robust molecular and phenotypic changes characteristic of EMT in NMuMG mouse mammary epithelial cells and A549 human lung cancer cells [40]. We treated NMuMG cells with TGFβ and performed RT-PCR analysis (primers in Table 1). We found that WWC1 expression was substantially downregulated (Figure 1C). We previously established an EMT cell model, DCIS-Sna-ER, which stably expressed inducible SNAI1 in MCF10DCIS.com transformed human basal breast epithelial cells [41]. Induction of SNAI1 potently repressed the expression of WWC1 and the canonical epithelial marker CDH1 (E-cadherin) (Figure 1D). The results demonstrate WWC1 downregulation in two EMT models. Since WWC1 was repressed by EMT, we investigated whether its expression correlated with epithelial/mesenchymal markers in 1673 established human cell lines in the DepMap datasets. Expression of WWC1 strongly correlated with epithelial markers CDH1 and EPCAM, and inversely with ZEB1/2 (Figure 1E), suggesting that WWC1 is highly expressed in epithelial cells and downregulated in EMT/mesenchymal cells. Collectively, the results suggest that WWC1 is transcriptionally repressed by EMT. As WWC1 is a direct target of EMT-TFs, its downregulation is an inherent feature of EMT.

### 3.2. EMT Blocks LATS and Causes Constitutive Activation of YAP

**Figure 2:**
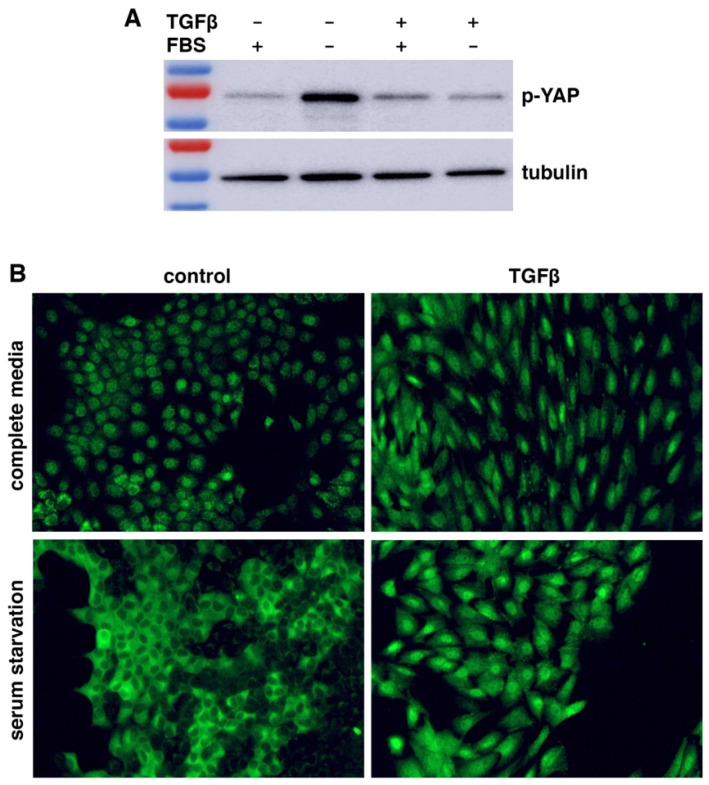
EMT prevents serum starvation-induced YAP phosphorylation and nuclear export. (**A**) EMT suppresses LATS-mediated YAP phosphorylation under serum starvation. NMuMG cells were treated with TGFβ (5 ng/mL) or mock-treated for 2 days, then placed in serum-free (FBS −) media or complete (FBS +) media for 8 h, followed by Western blotting with a Phospho-YAP (Ser127) antibody. β-tubulin served as a loading control. (**B**) YAP is constitutively localized in the nucleus in cells that have undergone EMT. NMuMG cells were treated with TGFβ (5 ng/mL) or mock-treated (control) for 2 days, then subjected to serum starvation for 1 day, followed by immunostaining with an anti-YAP antibody (green). The uncropped blots are shown in File S1.

WWC1 is important for the activation of Hippo signaling. Loss or depletion of WWC1 inhibits the LATS kinase, leading to hypo-phosphorylation and hence activation of YAP. Because EMT markedly downregulated WWC1, we asked if EMT could activate YAP. Serum contains lysophosphatidic acid that inhibits LATS, and serum starvation activates LATS to phosphorylate YAP [42]. We treated NMuMG cells with or without TGFβ for EMT induction, followed by serum starvation. Western blotting analysis with antibodies recognizing YAP Ser127 phosphorylation (a LATS phosphorylation site) showed that YAP was barely phosphorylated in NMuMG cells in regular media, indicative of its (partial) activation status (Figure 2A). When NMuMG cells were serum-starved, YAP became strongly phosphorylated (Figure 2A), demonstrating that serum starvation potently activates LATS. By contrast, when TGFβ-treated NMuMG cells were under serum starvation, YAP remained hypo-phosphorylated (Figure 2A). The results suggest that TGFβ-induced EMT prevents LATS activation by serum starvation.

LATS-mediated phosphorylation promotes cytoplasmic retention of YAP. We investigated YAP subcellular localization in EMT cells under serum starvation. When NMuMG cells were cultured in regular media, YAP was mostly nuclear (Figure 2B). Upon serum starvation, YAP was mostly exported to the cytoplasm (Figure 2B), consistent with LATS activation and YAP phosphorylation. TGFβ treatment induced EMT-like morphological changes (e.g., cell elongation), and YAP was localized in the nucleus (Figure 2B). However, when the TGFβ-treated NMuMG cells were under serum starvation, YAP still remained primarily nuclear (Figure 2B). The YAP subcellular localization patterns are consistent with its phosphorylation states (Figure 2A). Taken together, the results suggest that LATS kinase activation by stress signals is blocked in cells having undergone EMT, resulting in constitutive YAP hypo-phosphorylation and nuclear localization even under stress conditions.

### 3.3. EMT Activates YAP Target Gene Expression

**Figure 3:**
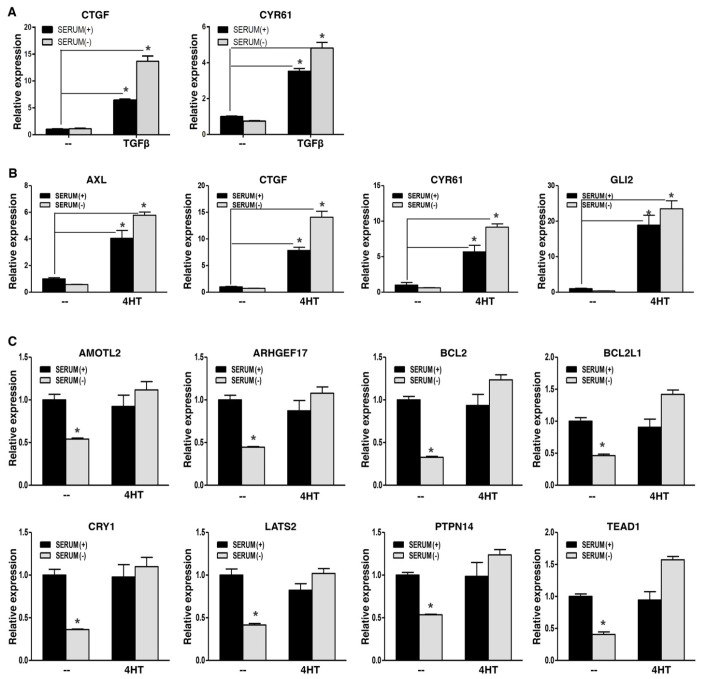
EMT activates YAP target gene expression. (**A**) Induction of CTGF and CYR61 by TGFβ. NMuMG cells were treated with TGFβ (5 ng/mL) or mock-treated (--) for 2 days, then subjected to serum starvation [serum (−)] for 8 h, followed by RT-PCR analysis. (**B**) Induction of selected YAP target genes by SNAI1-induced EMT. DCIS-Sna-ER cells were cultured in media containing 4HT (100 nM) or mock-treated for over 2 weeks, followed by serum starvation for 8 h. Expression of indicated YAP targets was determined by RT-PCR. (**C**) EMT sustains YAP target gene expression under serum starvation. In DCIS-Sna-ER cells (as described in (**B**)), expression of selected YAP target genes was measured by RT-PCR. Data shown as mean ± S.D. * *p* < 0.05.

WWC1 is an upstream activator of Hippo signaling and is required to inhibit YAP activity. EMT potently downregulated WWC1 expression, and led to persistent hypo-phosphorylation and nuclear localization of YAP. Active YAP acts as a transcriptional coactivator for the TEAD transcription factors to activate target gene expression. YAP and TAZ form liquid-liquid phase-separated condensates to promote gene transcription [43,44,45]. Nuclear localization alone does not indicate their full transcriptional activation. Therefore, we determined if EMT could upregulate YAP target gene expression. In a TGFβ-induced EMT model, YAP signature targets CTGF and CYR61 exhibited low basal expression in mouse NMuMG cells in regular media, despite nuclear localization of YAP; upon EMT induction by TGFβ, their expression was strongly upregulated (Figure 3A). When the EMT cells were under serum starvation, expression of CTGF and CYR61 was not downregulated and remained high (Figure 3A). Similarly, in an SNAI1-induced EMT model, EMT markedly upregulated representative YAP target genes, including AXL, CTGF, CYR61, and GLI2, in human breast carcinoma cells in media with or without serum (Figure 3B). Interestingly, for another group of YAP target genes (e.g., AMOTL2, anti-apoptotic BCL2 and BCL2L1), their basal expression was quite high, and serum starvation significantly repressed their expression (Figure 3C), suggesting that their basal expression is attributed to YAP activation, and Hippo signaling is intact and responsive to serum starvation. Following EMT induction, expression of these genes did not increase, but was sustained when cells were further under serum starvation (Figure 3C), suggesting that EMT totally blocks serum starvation-stimulated LATS activation. Overall, the results support that EMT prevents LATS activation, leading to constitutive YAP activation and high expression of YAP target genes. In line with this notion, expression of CTGF and CYR61 strongly correlated with mesenchymal markers in 1673 human cell lines (Figure S1). Together, the results suggest that EMT potently activates YAP target gene expression.

### 3.4. Repression of WWC1 Is Key to Activating YAP by EMT

**Figure 4:**
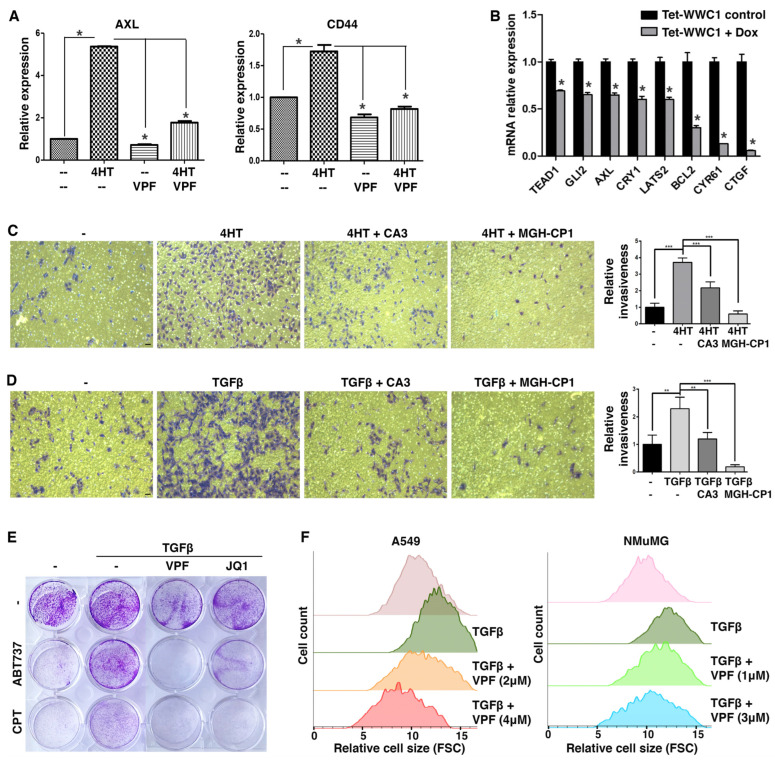
YAP inhibition suppresses EMT-induced YAP target gene expression, cell migration and invasion, chemoresistance, and cell size increase. (**A**) YAP inhibition blocks EMT-induced expression of AXL and CD44. DCIS-Sna-ER cells were treated without (--) or with 4HT (as described in Figure 3B), followed by treatment with verteporfin (VPF, 5 μM) or vehicle (DMSO, --) for 1 day. Expression of AXL and CD44 was determined by RT-PCR. (**B**) Restoration of WWC1 expression in mesenchymal Hs578T breast cancer cells represses YAP target gene expression. Hs578T cells were infected with lentivirus to stably express Tet-inducible WWC1. Following induction with Doxycycline (Dox), cells were subjected to RT-PCR analysis of the indicated YAP target genes. (**C**) TEAD-YAP inhibitors suppress EMT-stimulated cell migration in a Transwell-based assay. DCIS-Sna-ER cells were treated with 4HT or vehicle (DMSO, -) for 2 days, and plated in the Transwell inserts (with 8 μm pores) in serum-free media containing 4HT and CA3 (4 μM) or MGH-CP1 (10 μM). The inserts were placed into the bottom chambers containing complete media and incubated for 24 h. Cells that migrated through the membrane were stained, imaged, and quantified. Scale bar: 20 μm. (**D**) TEAD-YAP inhibition blocks TGFβ-induced cell invasion. A549 cells were treated with TGFβ (5 ng/mL) or mock-treated for 2 days, then plated in serum-free media containing TGFβ and CA3 (4 μM) or MGH-CP1 (10 μM) in the Transwell inserts that were pre-coated with a layer of Matrigel. The inserts were placed in the bottom chambers with complete media and incubated for 24 h. Cells that invaded the Matrigel and migrated through the membrane were stained and quantified. Scale bar: 20 μm. (**E**) YAP inhibition overcomes EMT-mediated apoptosis resistance. A549 cells in 12-well plates were treated with TGFβ (5 ng/mL) or mock-treated for 2 days, then cultured in serum-free media containing ABT737 (1 μM), camptothecin (CPT, 2.5 μM), VPF (10 μM), and/or JQ1 (1 μM) for 1 day. Cells were stained with crystal violet. (**F**) Verteporfin suppresses TGFβ-induced cell size growth. A549) and NMuMG cells were treated with TGFβ (5 ng/mL) or mock-treated for 2 days, and further treated with VPF or vehicle for 1 day. Cell sizes were determined by flow cytometry of forward scatter (FSC) measurement. Data shown as mean ± S.D. * *p* < 0.05. ** *p* < 0.01. *** *p* < 0.001.

We first took a pharmacological approach to verify that elevated expression of YAP target genes during EMT was indeed due to YAP activation. In the SNAI1-induced EMT model, upregulation of AXL during EMT was largely abrogated by treatment with TEAD-YAP inhibitor verteporfin [46] (Figure 4A). CD44 is often considered a cancer stem cell marker, and its expression is induced by EMT [47]. CD44 was recently identified as a direct target gene of YAP [48]. Upregulation of CD44 during EMT was also blocked by verteporfin (Figure 4A). The results suggest that YAP activation causes the upregulation of its target genes during EMT.

Proper Hippo signaling requires intact apical cell junctions and polarity [49,50], which are part of epithelial characteristics. Such epithelial structures are disrupted during EMT, thus presumably impairing Hippo signaling and activating YAP. We next asked if repression of WWC1 was critical for YAP activation by EMT. The highly mesenchymal Hs578T TNBC cells were often used as a stable EMT cell model. Based on DepMap gene expression, Hs578T cells exhibit very little expression of WWC1 but high expression of many YAP target genes (indicative of YAP activation). We infected Hs578T cells with lentiviruses inducibly expressing exogenous WWC1. Induction of WWC1 did not cause evident morphological changes (i.e., mesenchymal-to-epithelial transition), but substantially downregulated many YAP target genes (e.g., CTGF, CYR61) (Figure 4B). Taken together, the results suggest that induction of YAP target genes in EMT/mesenchymal cells is largely mediated by active YAP, and repression of WWC1 expression is a key mechanism underlying YAP activation by EMT.

### 3.5. EMT-Stimulated Cellular Phenotypes Are Attributed to YAP Activation

Both EMT and YAP drive many overlapping cellular phenotypes. As EMT activates YAP transcriptional function, we determined if EMT-stimulated cell motility and invasiveness were attributed to YAP activation. In a Transwell-based cell migration assay, SNAI1-driven EMT boosted cell migration through a porous membrane, which, however was substantially suppressed by treatment with TEAD-YAP inhibitor CA3 [51] or MGH-CP1 [52] (Figure 4C). In a Transwell-based cell invasion assay in which the porous membrane was pre-coated with a layer of extracellular matrix (ECM) protein mixture, TGFβ stimulated A549 lung cancer cell invasion through ECM, and treatment with CA3 or MGH-CP1 suppressed the pro-invasion effects of TGFβ (Figure 4D). The results suggest that EMT-enhanced cell motility and invasiveness are dependent on YAP activation.

EMT confers resistance to apoptosis and certain chemotherapy drugs, whereas YAP is known to activate anti-apoptotic genes and promote cell survival. Therefore, we tested if EMT-conferred chemoresistance was mediated by YAP. ABT-737 is a potent BH3 mimetic inhibitor of BCL2 and BCL2L1, and induces apoptosis [53,54,55]. Camptothecin is a topoisomerase I inhibitor that causes DNA damage and apoptosis [56], and its cytotoxicity is suppressed by increased BCL2 expression [57]. Under serum starvation conditions, A549 cancer cells were sensitive to ABT-737 and camptothecin (Figure 4E). Treatment with TGFβ rendered A549 cells resistant to these compounds (Figure 4E). However, co-treatment with verteporfin re-sensitized these cells to ABT-737 and camptothecin (Figure 4E). The transcriptional activity of YAP depends on the bromodomain protein BRD4, a co-activator [58]. Treatment with JQ1, a small-molecule inhibitor of BRD4 [59], also overcame TGFβ-induced resistance to ABT-737 and camptothecin (Figure 4E). The results suggest that YAP critically contributes to EMT-induced apoptosis resistance and chemoresistance.

It was previously reported that TGFβ-induced EMT led to increased cell size and cellular protein content by activation of the mTOR pathway [60]. There exists extensive crosstalk between the mTOR and Hippo pathways, and YAP can activate mTOR [61]. Therefore, we investigated whether EMT increased cell sizes via YAP. Following TGFβ treatment, A549 and NMuMG cells became enlarged (Figure 4F). Verteporfin treatment reversed the cell size increases in a dose-dependent manner (Figure 4F), suggesting that YAP activity is required for EMT-induced cell size growth.

We further validated that suppression of EMT-stimulated cellular phenotypes by pharmacological inhibition of YAP was not due to possible reversal of EMT. When NMuMG and A549 cells were treated with TGFβ, typical EMT-like morphological changes were observed, and the epithelial marker CDH1 was downregulated (Figure S2). When these EMT cells were further treated with various TEAD-YAP inhibitors for one day, they maintained the mesenchymal-like morphology, and CDH1 levels were not restored (Figure S2), suggesting that under the experimental settings, pharmacological inhibition of TEAD-YAP does not overtly reverse TGFβ-driven EMT. Therefore, pharmacological blockade of TEAD-YAP suppresses EMT-associated cellular phenotypes without reversing the EMT state.

### 3.6. YAP Activation Induces Immune Checkpoints and Suppresses T Cell Effector Function

**Figure 5:**
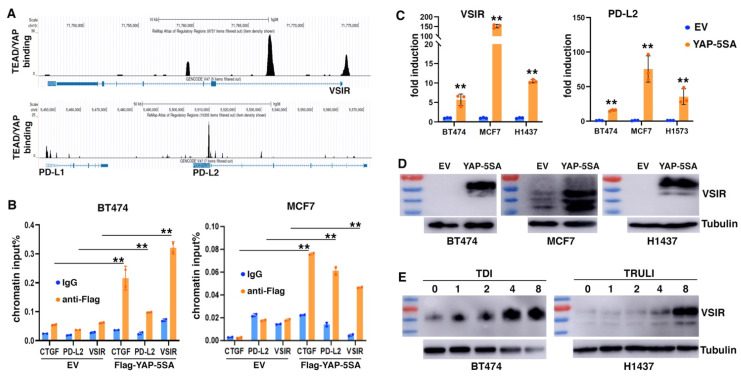
YAP activation directly induces expression of PD-L2 and VSIR. (**A**) ReMap analysis of TEAD-YAP genomic binding at VSIR, PD-L2 (PDCD1LG2), and PD-L1 (CD274). (**B**) Validation of YAP genomic binding at VSIR and PD-L2. BT474 and MCF7 cells were transduced with lentiviral Flag-tagged YAP-5SA or empty vector (EV), and subjected to ChIP analysis with anti-Flag antibodies or IgG antibody control. (**C**) RT-PCR analysis showing that active YAP induced VSIR and PD-L2. (**D**) Immunoblotting analysis showing induction of VSIR proteins by YAP activation in various cell lines. VSIR is less glycosylated in MCF7 cells. (**E**) Pharmacological inhibition of LATS kinases induces VSIR expression. BT474 and H1437 cells were treated with LATS inhibitors at indicated concentrations (in μM), followed by immunoblotting with anti-VISR antibodies. Data shown as mean ± S.D. ** *p* < 0.01. The uncropped blots are shown in File S1.

Immune suppression is increasingly recognized as a critical EMT function. We asked if YAP activation might contribute to EMT-mediated impact on tumor immune responses. We mined the ChIP-seq data of YAP and TEAD4 in MDA-MB-231 cells (SRA: SRP055170; GEO: GSE66081) [62] and identified their evident genomic binding at two members of the B7 family of immune checkpoints [37]: VISTA (VSIR) and PD-L2 (PDCD1LG2). ReMap ChIP-seq data confirmed the strong binding of TEAD1/4 and YAP at the promoter and intron 1 of VSIR, as well as at the promoter of PD-L2 in a variety of cancer cells (Figure 5A). The TEAD binding consensus sequence (5′-CATTCC) was found at these peaks. Weak binding of TEAD and YAP was also found at the 5′ region of PD-L1 (CD274) (Figure 5A), which was previously reported as a YAP/TAZ target gene [32,33,34,35,36]. To validate YAP binding at VSIR and PD-L2, we transduced BT474 and MCF7 epithelial breast cancer cells with lentiviruses expressing Flag-tagged YAP-5SA (an active form of YAP) [63], and performed a ChIP assay with anti-Flag antibodies or an IgG antibody control (PCR primers in Table 2). Significant binding of Flag-YAP was detected at CTGF (positive control), VSIR, and PD-L2 (Figure 5B), confirming that VSIR and PD-L2 are direct genomic target genes of YAP.

We determined if YAP activation could induce expression of VSIR and PD-L2. Based on DepMap gene expression data of YAP targets CTGF and CYR61, we selected a few cell lines that exhibit low YAP activity. Overexpression of YAP-5SA in MCF7 and BT474 cells, as well as H1437 and H1573 lung cancer cells, robustly upregulated VSIR and PD-L2 RNA expression (Figure 5C). We further verified VSIR induction by YAP-5SA by immunoblotting. Human VSIR protein has a predicted molecular weight around 34 kDa, but migrates at 55–70 kDa in SDS-PAGE due to glycosylation [64]. There was little basal expression of VSIR in BT474, MCF7, and H1437 cells, but transduction with YAP-5SA markedly induced VSIR protein expression (Figure 5D). The induced VSIR proteins were highly glycosylated (~70 kDa) in BT474 and H1437 cells, while less glycosylated in MCF7 cells (Figure 5D). Moreover, we investigated potential VSIR induction by activation of endogenous YAP. TDI-011536 [65] and TRULI [66] are related potent LATS inhibitors, and both prevent YAP phosphorylation by LATS. We treated BT474 and H1437 cells with these compounds and observed dose-dependent induction of VSIR (Figure 5E). The results suggest that YAP activation directly induces VSIR and PD-L2 expression.

We next validated whether expression of VSIR and PD-L2 correlated with YAP activity in human cancer cell lines. While genetic alterations in Hippo signaling are rare in human cancer, several pathway components, particularly NF2, are frequently inactivated by somatic mutations in malignant mesothelioma, leading to common YAP/TAZ activation in this rare type of cancer [67]. According to DepMap gene expression data, the vast majority of human cancer cell lines express low levels of PD-L2, but most mesothelioma cell lines exhibit fair expression of PD-L2 (Figure S3A). VSIR is also strongly expressed in most mesothelioma cell lines (Figure S3B). Moreover, expression of PD-L2 and VSIR significantly correlated with that of YAP signature target genes (Figure S3C,D). The data are consistent with YAP-driven induction of PD-L2 and VSIR.

**Figure 6:**
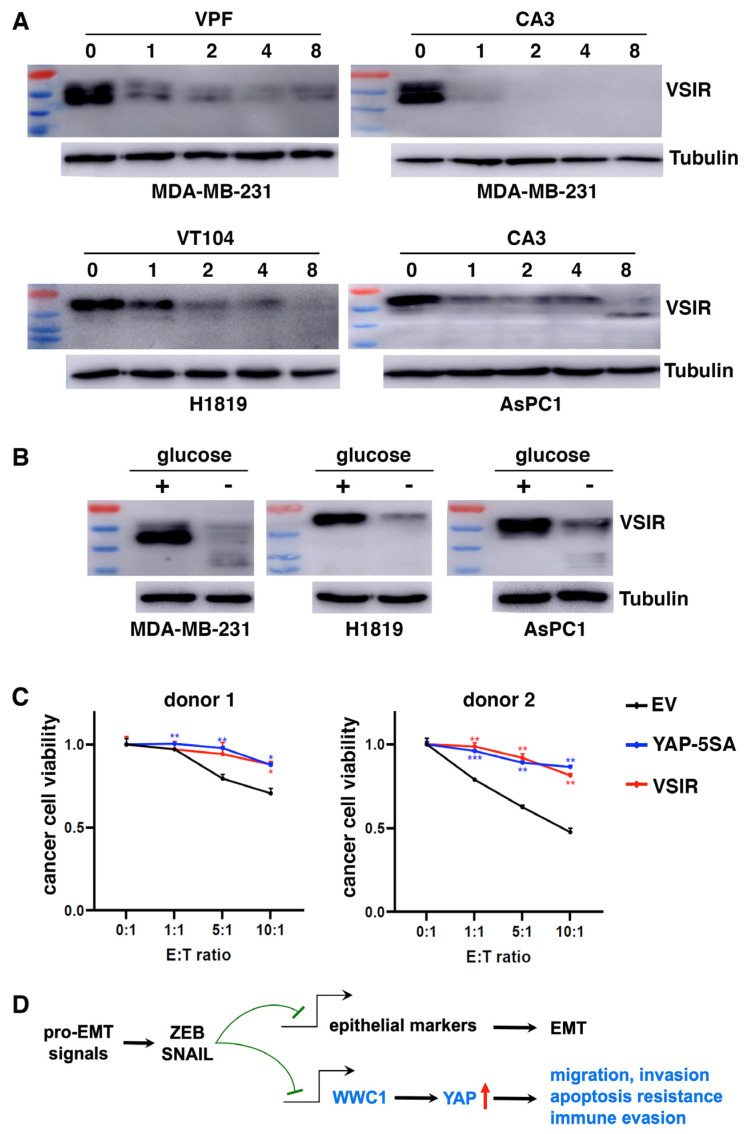
YAP activation is required for VSIR expression and confers resistance to T cell cytotoxicity. (**A**) Pharmacological inhibition of YAP decreases VSIR expression. Carcinoma cells were treated with TEAD-YAP inhibitors at indicated concentrations (in μM), followed by immunoblotting of VSIR. (**B**) Glucose starvation downregulates VSIR expression. Cancer cells were cultured in regular or glucose-free media overnight and subjected to immunoblotting of VSIR. (**C**) YAP activation or VSIR overexpression confers resistance to cytotoxic CD8 T cells. BT474 cells were transduced with lentiviral empty vector (EV), YAP-5SA or VSIR, and incubated with an increasing number of activated CD8+ T cells from two donors. Viable cancer cells were counted. E: effector T cells; T: target tumor cells. * *p* < 0.05, ** *p* < 0.01, *** *p* < 0.001. (**D**) EMT activates YAP to promote multiple malignant phenotypes: EMT-TFs ZEB and SNAIL directly repress not only a multitude of epithelial genes to shed epithelial traits, but also additional genomic targets, including WWC1. Repression of WWC1 impairs Hippo signaling and aberrantly activates YAP, which drives malignant progression and immune evasion. The uncropped blots are shown in File S1.

We further determined if high VSIR expression in certain cancer cells was driven by endogenous YAP. Consistent with YAP activation by EMT, MDA-MB-231 mesenchymal cancer cells exhibited high basal expression of VSIR proteins (Figure 6A). H1819 lung cancer and AsPC1 pancreatic cancer cells also exhibited high YAP activity (evidenced by high expression of YAP target genes in DepMap) and high VSIR expression (Figure 6A). We treated these cells with TEAD-YAP inhibitors verteporfin, CA3, and VT104 [68]. YAP inhibition downregulated VSIR in a dose-dependent manner (Figure 6A). In addition to pharmacological inhibition of YAP, energy stress such as glucose starvation activates both LATS and AMP-activated protein kinase (AMPK), which inactivate YAP by phosphorylation [69,70]. When cultured in glucose-free media, MDA-MB-231, H1819, and AsPC1 cells all showed markedly downregulated VSIR protein expression (Figure 6B). These results suggest that YAP activation is required for high VSIR expression in mesenchymal or YAP-activated cancer cells.

As YAP activation induced multiple immune checkpoints, we asked if it conferred resistance to CD8^+^ T cell-mediated cytotoxicity. We co-cultured increasing numbers of activated human HLA-A2^−^ CD8^+^ T cells with control, YAP-5SA- or VSIR-overexpressing BT474 cancer cells. YAP activation or VSIR overexpression increased cancer cells’ resistance to T cell-mediated destruction (Figure 6C). Taken together, YAP activation induces immune checkpoints and enables cancer cells to resist effector T cells.

## 4. Discussion

### 4.1. EMT Represents a Non-Genetic Mechanism Activating YAP in Cancer

YAP is pervasively activated in human cancer; however, genetic alterations in the core components of the Hippo signaling pathway are uncommon. WWC1 is a scaffold protein that integrates multiple Hippo pathway components into biological complexes/condensates, and is critical for the activation of Hippo signaling. In this study, WWC1 is identified as a direct transcriptional target of EMT-TFs. EMT inherently represses WWC1 expression, thus deactivating Hippo signaling and constitutively activating YAP even in the absence of mutations. Because restoration of WWC1 in mesenchymal cells by exogenous expression was sufficient to inactivate YAP (Figure 4B), downregulation of WWC1 is a critical mechanism by which EMT activates YAP. Therefore, EMT represents a non-genetic mechanism underlying YAP activation. As the occurrence of EMT is prevalent in human carcinomas [2], most tumors are expected to contain a subpopulation of malignant cells that exhibit EMT and YAP activation. It was previously reported that overexpression of YAP/TAZ or knockdown of WWC1 promoted EMT [71,72,73,74,75]. Therefore, EMT can be induced by diverse signaling events (inactivation of Hippo signaling is one of them), and following EMT, YAP is activated and, in turn, maintains the EMT state by a feed-forward loop.

### 4.2. YAP Mediates EMT-Stimulated Malignant Phenotypes

EMT is accompanied by the acquisition of manifold malignant phenotypic changes in cancer cells, but the underlying molecular mechanisms remain incompletely understood. It has been challenging to directly target EMT or simultaneously target its diverse phenotypes. Here, we uncovered that the EMT process intrinsically inhibits Hippo signaling, leading to constitutive YAP activation. The YAP-dependent transcriptional program promotes a variety of cellular traits that are strikingly similar to those induced during EMT. Indeed, we found that pharmacological inhibition of YAP largely abolished EMT-stimulated cell migration/invasion, apoptosis resistance, and cell size growth, without evident reversal of EMT, supporting that YAP is a key common mediator for EMT-associated diverse phenotypes (Figure 6D). Identification of a common molecular pathway underlying EMT-associated various malignant properties is desirable for developing new therapeutic approaches. TEAD-YAP inhibitors may effectively overcome EMT’s contributions to malignant progression and drug resistance.

### 4.3. The EMT-YAP Axis Drives Immune Evasion

Since the initial discovery of EMT’s role in immunosuppression [76], emerging evidence has revealed that EMT induces immune evasion through multiple mechanisms [77], including upregulation of PD-L1 expression [78]. Meanwhile, YAP/TAZ activation in human cancer cells directly induced PD-L1 expression, leading to T cell exhaustion [32,33,34,35,36]. This study further uncovered that activation of YAP induces expression of PD-L2 and VSIR. PD-L1/L2 are PD-1 ligands and targets of immune checkpoint blockade therapy. In adaptive immune response, tumor-engaged effector T cells secrete interferon-gamma (IFN-γ), which induces PD-L1/L2 expression on tumor cells [79,80,81]. While PD-L1 dampens T cell cytotoxicity, its expression in this scenario is also a surrogate marker of preexisting adaptive immunity. Therefore, tumor PD-L1 expression has often been used as one of the predictive biomarkers for sensitivity to immunotherapy targeting PD-1/PD-L1. However, PD-L1 as a predictive biomarker has limitations. An analysis of FDA-approved immunotherapies found that PD-L1 was predictive in less than 30% of cases [82]. Because PD-L1 can be induced by diverse mechanisms other than IFN-γ signaling, its expression driven by oncogenic pathways (e.g., YAP activation) does not reflect preexisting T cell immunity and is not a suitable biomarker for immunotherapy response.

VSIR is also a B7 family immune checkpoint molecule expressed in multiple immune cell types (especially those of the myeloid lineage) and tumor cells [83]. DepMap gene expression data show that among a large panel of established human cell lines, VSIR is broadly expressed in human cancer cells, including various types of solid tumors. VSIR expression in tumor cells suppresses T cell proliferation in vitro and tumor infiltration in vivo [84]. Several VSIR-binding proteins have been reported. Recently, LRIG1 was identified as an inhibitory receptor for VSIR to suppress T cell immune responses [85]. LRIG1 is highly expressed on activated tumor-specific CD8+ T cells, and the VSIR-LRIG1 signaling axis inhibits T cell proliferation, survival, and effector function [85]. Overall, YAP may induce multiple immune checkpoint proteins in cancer cells to inhibit T cell antitumor activity and enable immune escape. Of note, induction of such immune checkpoints by YAP may be context/species-dependent. We transduced several murine carcinoma cell lines with lentiviral YAP-5SA but failed to induce these immune checkpoint proteins. It was reported previously that YAP activation unexpectedly enhanced anti-tumor immunity [24]; it is possible that LATS1/2 deletion in mouse tumor cell models (B16, SCC7, and 4T1) in that study did not upregulate the immune checkpoints.

Immune checkpoint inhibitors (ICIs) targeting PD-1/PD-L1 and CTLA-4 improve antitumor T cell responses and patient survival. However, the overall response rate to these immunotherapies is still low. YAP-activated human tumor cells likely resist anti-PD-1/PD-L1 therapy due to the expression of additional immune checkpoint proteins. Indeed, combinatorial treatment with anti-VSIR and anti-PD-1 or PD-L1 antibodies achieved synergistic therapeutic efficacy in syngeneic murine tumor models [86,87,88,89]. Inclusion of VSIR blockade in combinatorial immunotherapies may help overcome resistance to current ICIs in YAP-activated cancer cells. The immune checkpoint inhibitor CA-170 has dual targeting activities against PD-L1/L2 and VSIR [90] and may be effective for targeting tumors with YAP activation. Administration of TEAD-YAP inhibitors downregulates immune checkpoint expression in YAP-activated tumors and may also enhance their responses to ICIs.

## 5. Conclusions

EMT promotes cancer metastasis, immune evasion, and therapy resistance. This study uncovered that the EMT process inherently represses WWC1 expression, leading to constitutive YAP activation. Given its prevalence, EMT may represent a non-genetic mechanism underlying pervasive YAP activation in human cancer. Our results further suggest that the YAP-driven transcriptional program critically contributes to various EMT-induced malignant phenotypes. Therefore, pharmacological targeting of YAP may abolish EMT-enhanced cancer properties and overcome resistance to cancer therapies, including immune checkpoint blockade. However, the study is limited to in vitro cell culture-based experimental assays. It remains to be determined if the findings can be validated in human cancers in vivo.

## Figures and Tables

**Table 1:** Primers for RT-PCR analysis of gene expression.

Gene	Forward Primer	Reverse Primer
Mouse β-actin	GTCGTCGACAACGGCTCC	TTCCCACCATCACACCCTGG
Mouse WWC1	AGTCGATGTCTGCACCACTG	GATTGTACCAGCGCGTTGAC
Mouse CTGF	ACCGCAAGATCGGAGTGTG	TCCAGGCAAGTGCATTGGT
Mouse CYR61	ACCCTTCTCCACTTGACCAG	TTAGCGCAGACCTTACAGCA
Human β-actin	GGATTCCTATGTGGGCGACGA	GCGTACAGGGATAGCACAGC
Human WWC1	CAGGTGCAGACAGGCAAAGAT	TGCCTGCCTTTGCTTGTAGA
Human CTGF	GCTTACCGACTGGAAGAC	ACTTGATAGGCTTGGAGATT
Human CYR61	AAGGGGCTGGAATGCAACTT	TTGGGGACACAGAGGAATGC
Human AXL	CAGAGGTGCTAATGGACATAG	CGGTGGACAAGGAAGAGAG
Human GLI2	GTTCGAGCAGCTCAAGAAGG	GGCTCAGCATGGTCACCTC
Human AMOTL2	AGGAGGCTGCAAGACTTCAA	CAGCTTCTCTTGCTCCTGCT
Human ARHGEF17	CCGCCTTGGTTTTGAACAGG	GCTGTTGCAGACCCATACCT
Human BCL2	CTTTGAGTTCGGTGGGGTCA	CCGTACAGTTCCACAAAGGC
Human BCL2L1	CTGACATCCCAGCTCCACAT	GTGGATGGTCAGTGTCTGGT
Human CRY1	CAGGTTGTAGCAGCAGTGGA	GACTAGGACGTTTCCCACCA
Human LATS2	TCATCCACCGAGACATCA	CCACACCGACAGTTAGAC
Human PTPN14	GTTCACGTCCAGTGTGGTGA	AGCAGTTGAGGGAGTTGACG
Human TEAD1	GATGATGCTGGGGCTTTTTA	GCCATTCTCAAACCTTGCAT
Human CD44	CCTGCCCAATGCCTTTGATG	CAGGGACTGTCTTCGTCTGG
Human CDH1	TTACTGCCCCCAGAGGATGA	TGCAACGTCGTTACGAGTCA
Human VSIR	CCCATCCTCCTCCCAGGATA	GCCGGGGTTTTCAATCCCTT
Human PD-L2	CAAGTGAGGGACGAAGGACAG	GACGTTTGGCCAGGATACTTCT

**Table 2:** Primers for ChIP PCR analysis.

Gene	Forward Primer	Reverse Primer
CTGF	CTCTTCGCACCACTCCTGAT	CAGTGGACAGAACAGGGCAA
PD-L2	TGTTCAAGCGATGGGACGAA	GATGTGGGGCTGAACACTCA
VSIR	CTAAGCTCACGCCCTGTCAT	CTGTGGCACCCTCAGATGTT

## Data Availability

The ChIP-seq data we analyzed were derived from the public SRA: https://www.ncbi.nlm.nih.gov/sra?term=ERP116911 and https://www.ncbi.nlm.nih.gov/sra?term=SRP055170; ReMap ChIP-seq assay was available at UCSC genome browser (https://genome.ucsc.edu/); Gene expression data in human cell lines were available from DepMap (https://depmap.org/portal/).

## Supplementary Materials

The following supporting information can be downloaded at: https://www.mdpi.com/article/10.3390/cancers17172767/s1, Figure S1: Correlations between expression of YAP target genes and EMT markers; Figure S2: Pharmacologic blockade of YAP-TEAD does not reverse TGFβ-induced EMT; Figure S3. Expression of PD-L2 and VSIR correlates with YAP activity in human cell lines; File S1: Western blot information.
