# Supplementary material for: Epithelial-Mesenchymal Transition Activates YAP to Drive Malignant Progression and Immune Evasion
Source: Cancers (Basel). 2025 Aug 25;17(17):2767. doi: 10.3390/cancers17172767 (PMC12427426; doi:10.3390/cancers17172767)

NMuMg Cell Samples:
serum-free medium treated for **8h;**
cell density reached **50%** before medium replacement

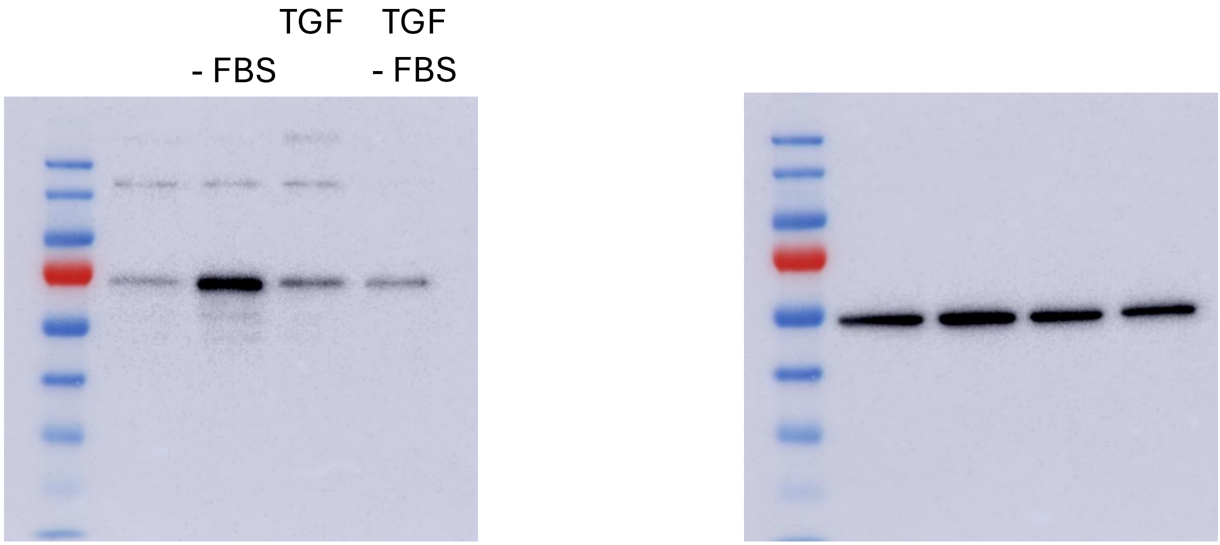

pYAP

Tubulin

YAP Overexpression Promotes VSIR Expression

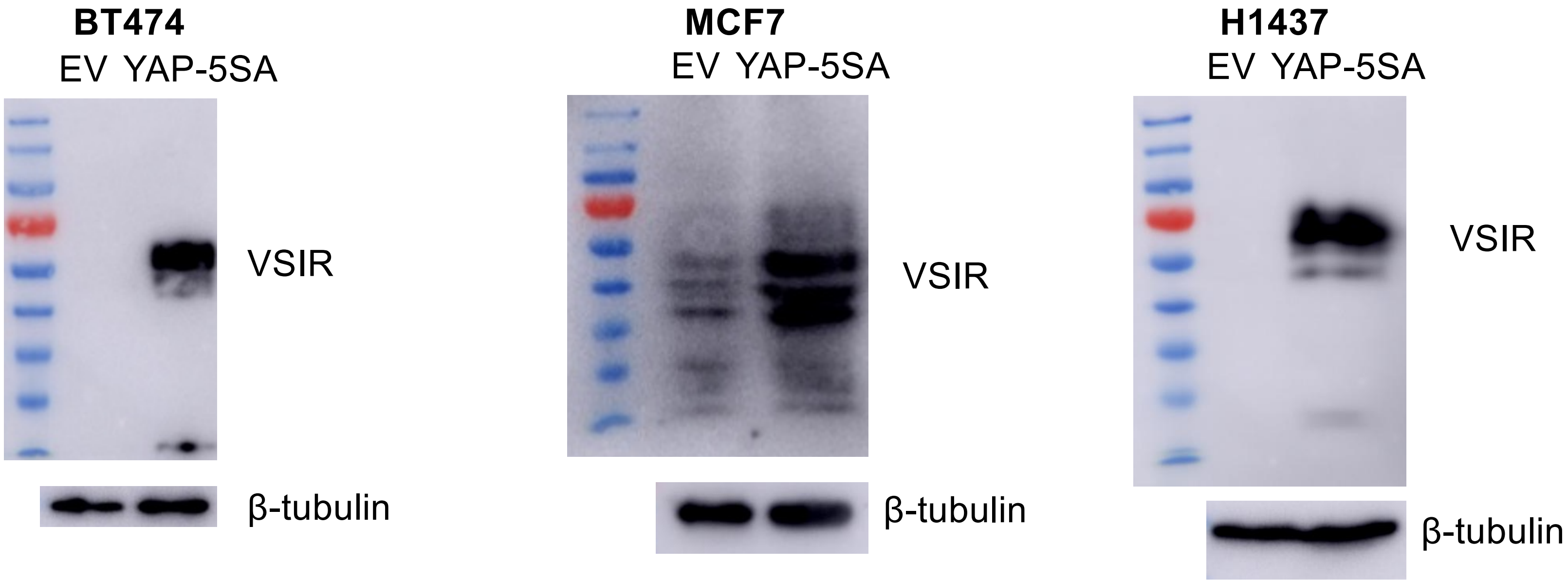

YAP Upregulation by LATS inhibitors Promotes VSIR Expression

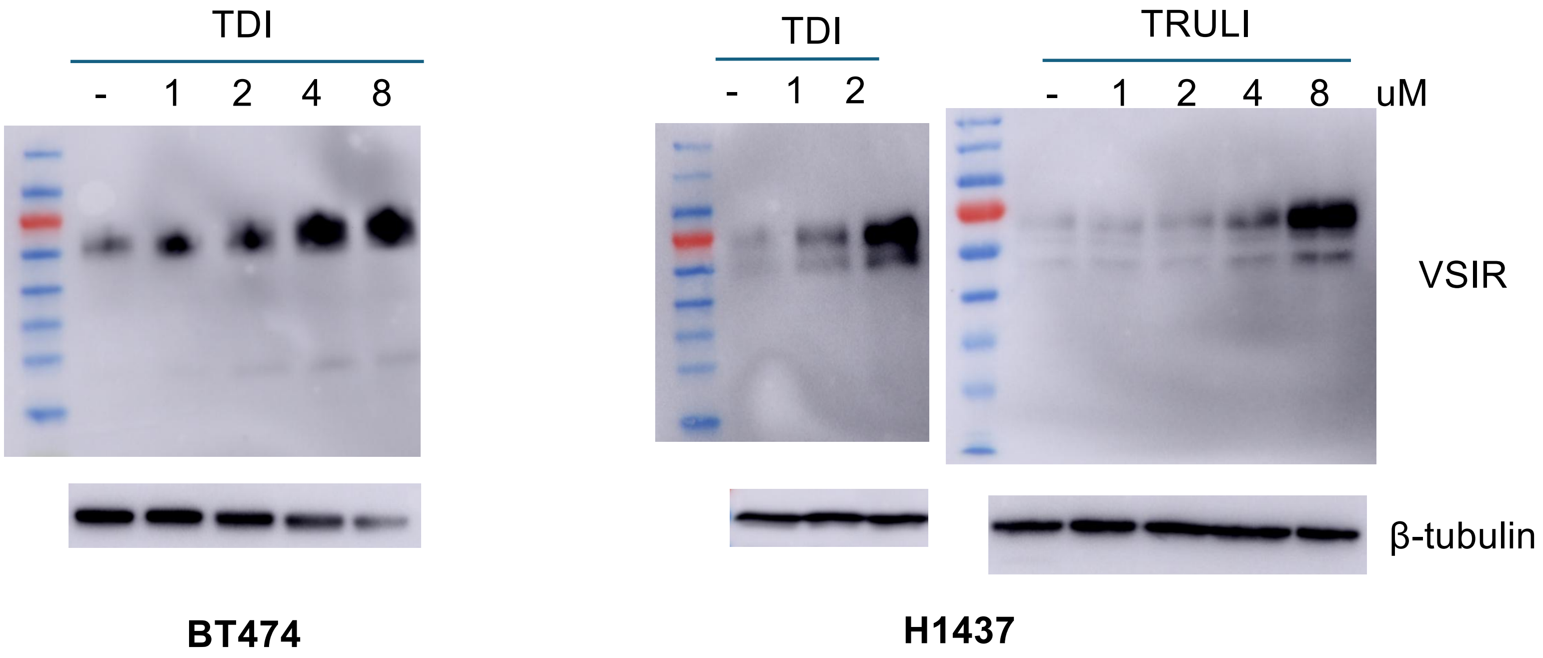

YAP-TEAD Inhibitors on Relative High YAP Cells Reduces VSIR Expression

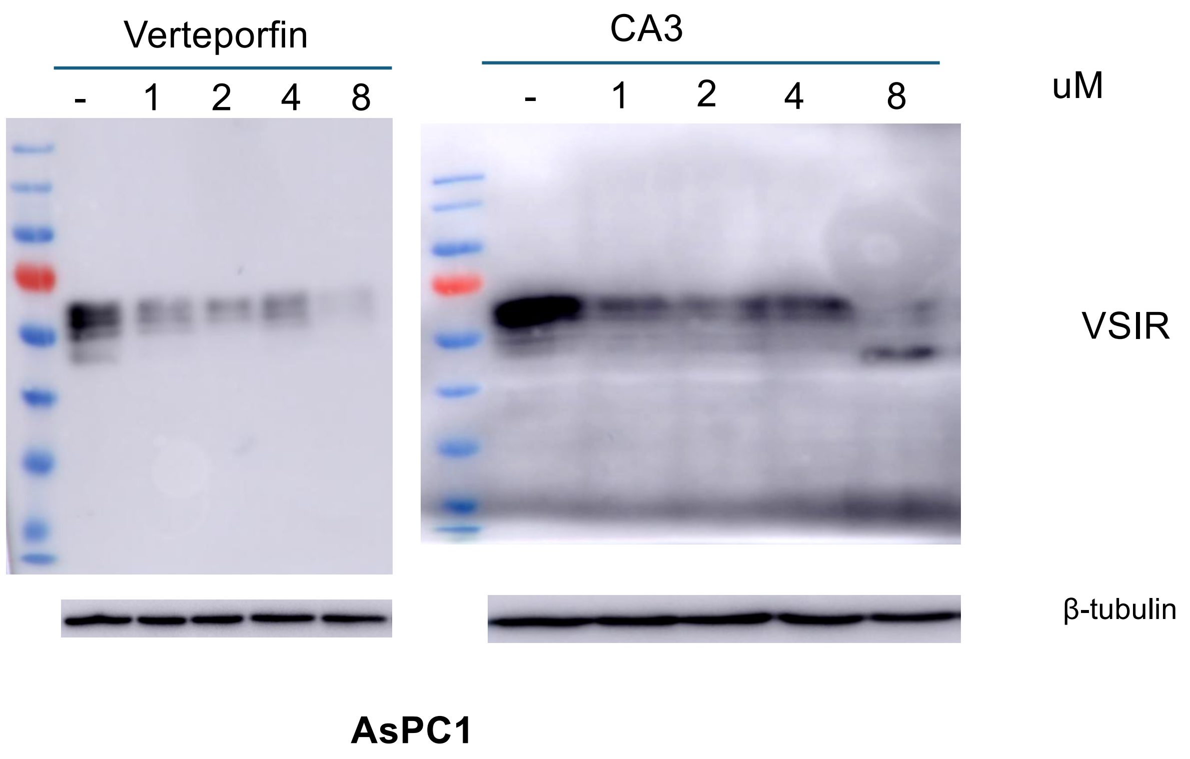

YAP-TEAD Inhibitors on Relative High YAP Cells Reduces VSIR Expression

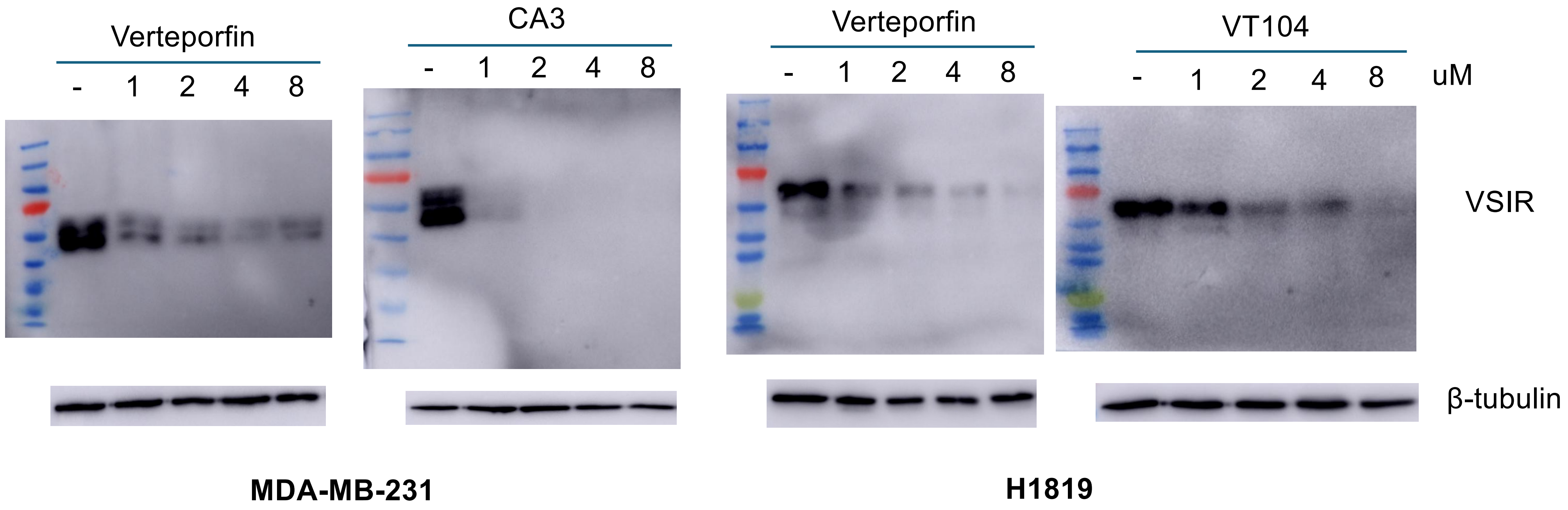

Glucose Starvation Induced YAP Downregulation Reduces VSIR Expression

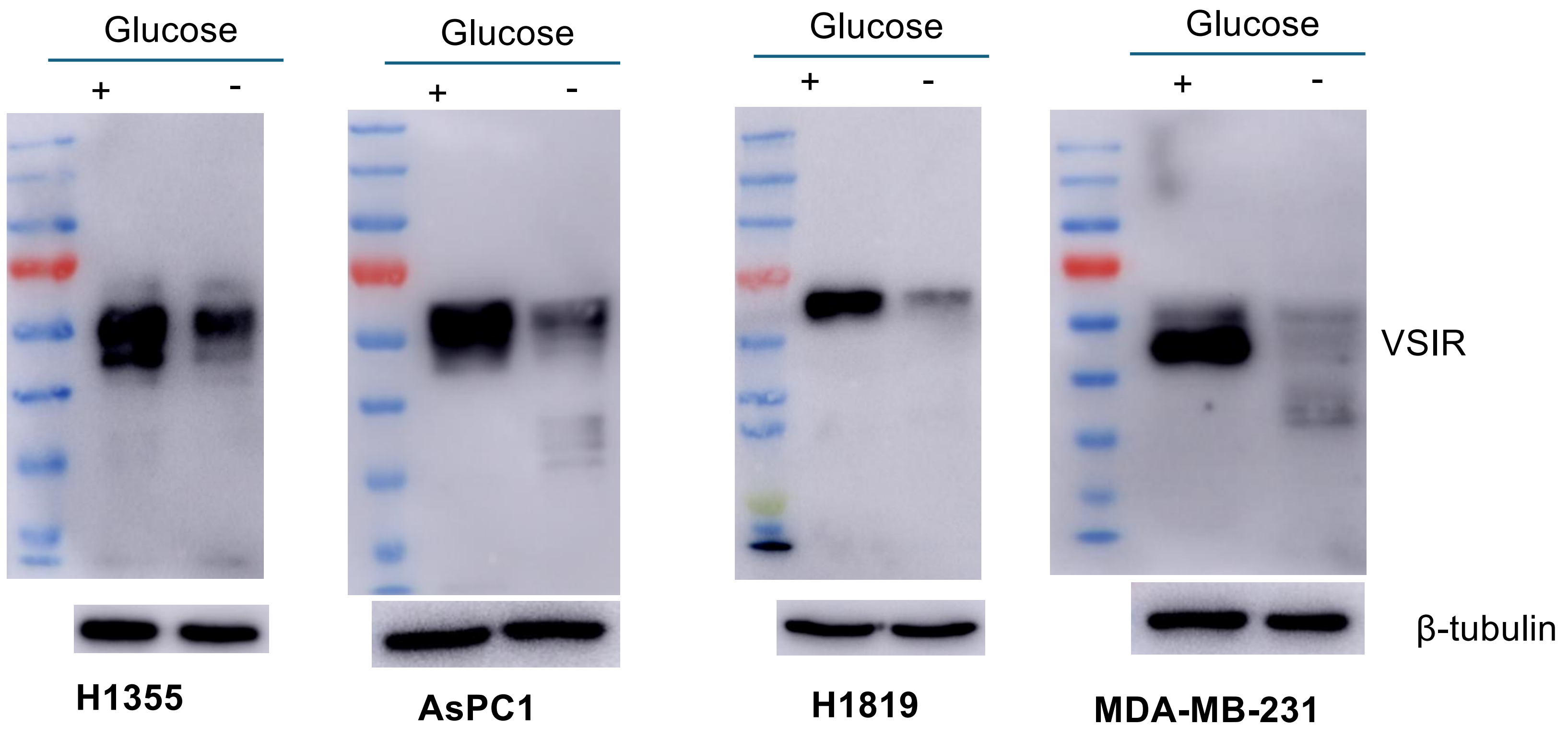

Usage of YAP Inhibitors Did Not Reverse EMT Progress.

Cells were treated w/n TGF-b for 2d, then added YAP inhibitors for 8h. Lyse cells for epithelial marker signal measurement.

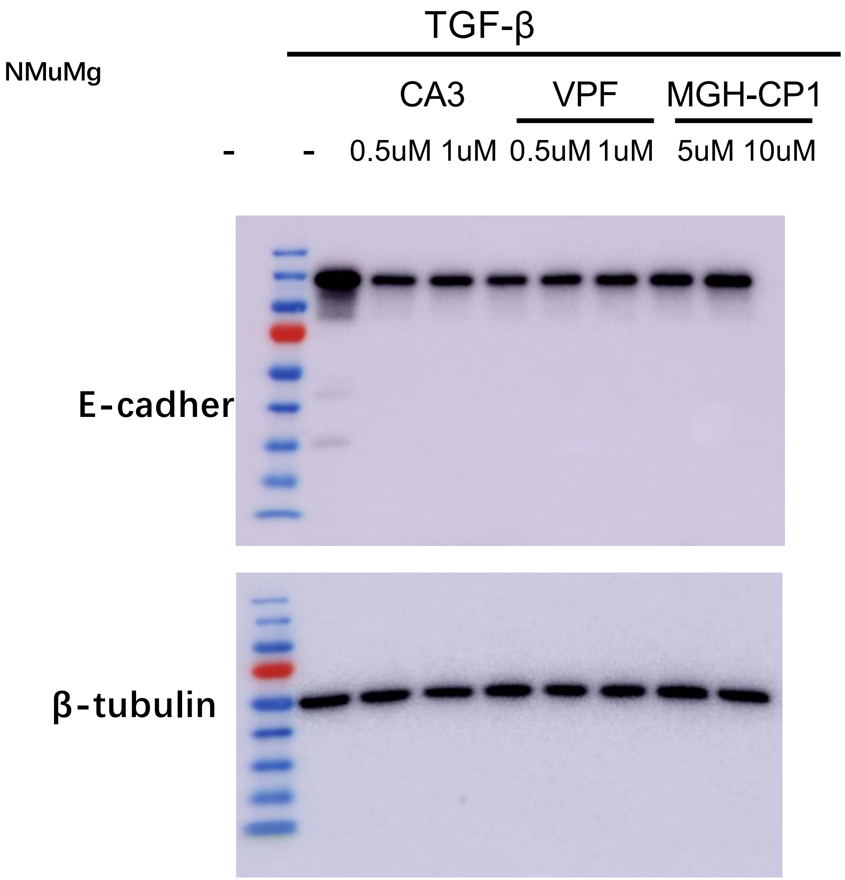

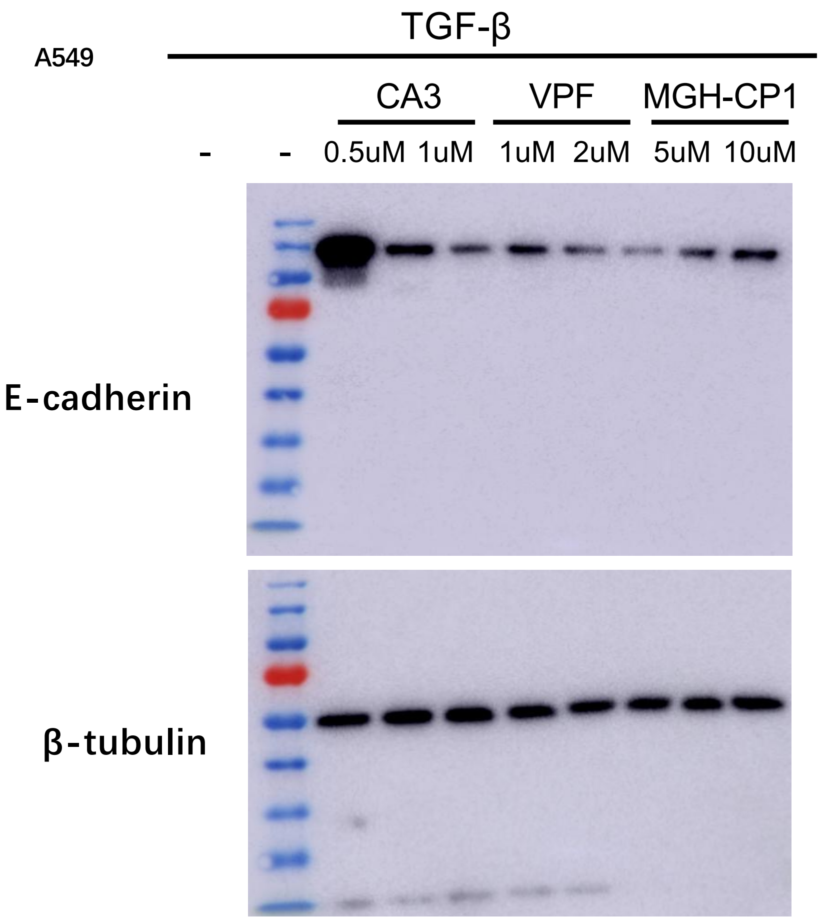

Supplement: Supplementary file 1 [file cancers-17-02767-s001.zip › File S1.pdf]
