# Supplementary material for: Epithelial-Mesenchymal Transition Activates YAP to Drive Malignant Progression and Immune Evasion
Source: Cancers (Basel). 2025 Aug 25;17(17):2767. doi: 10.3390/cancers17172767 (PMC12427426; doi:10.3390/cancers17172767)
Supplement: Supplementary file 1 [file cancers-17-02767-s001.zip › supplementary Figures.pdf]

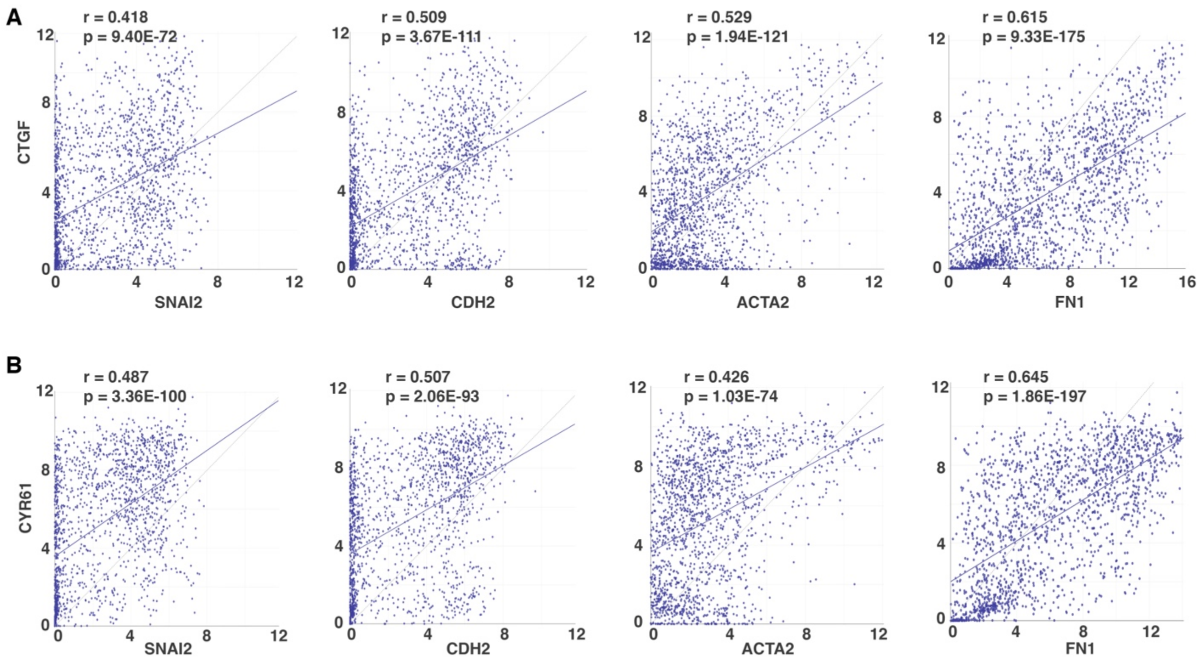

Figure S1. Correlations between expression of YAP target genes and EMT markers. DepMap gene expression analysis shows correlations between representative YAP target genes CTGF (**A**) and CYR61 (**B**) with indicated EMT/mesenchymal markers in 1,673 human cell lines. Linear regression lines, Pearson correlation (r), and p value of linear regression are shown. Each dot represents a cell line. The Y axis shows CTGF or CYR61 expression (log2), and the X axis shows expression (log2) of indicated EMT/mesenchymal markers.

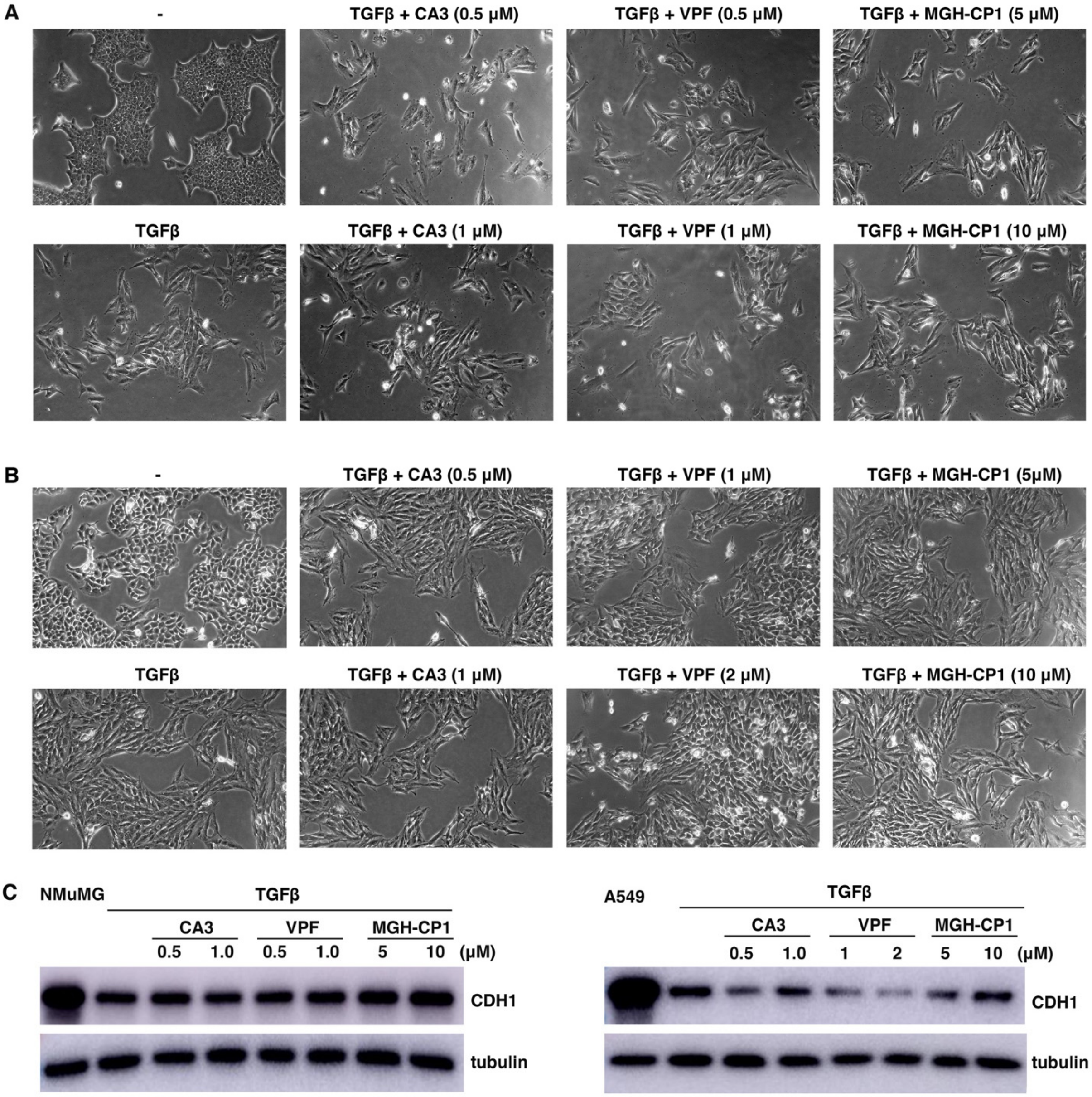

Figure S2. Pharmacologic blockade of YAP-TEAD does not reverse TGFβ-induced EMT. NMuMG (**A**) and A549 (**B**) cells were treated with TGFβ or mock treated for 2 days, then YAP-TEAD inhibitors were added to the cultures for 1 day. Bright-field images were captured, and cells were subjected to Western blotting analysis for E-cadherin (CDH1) expression (**C**).

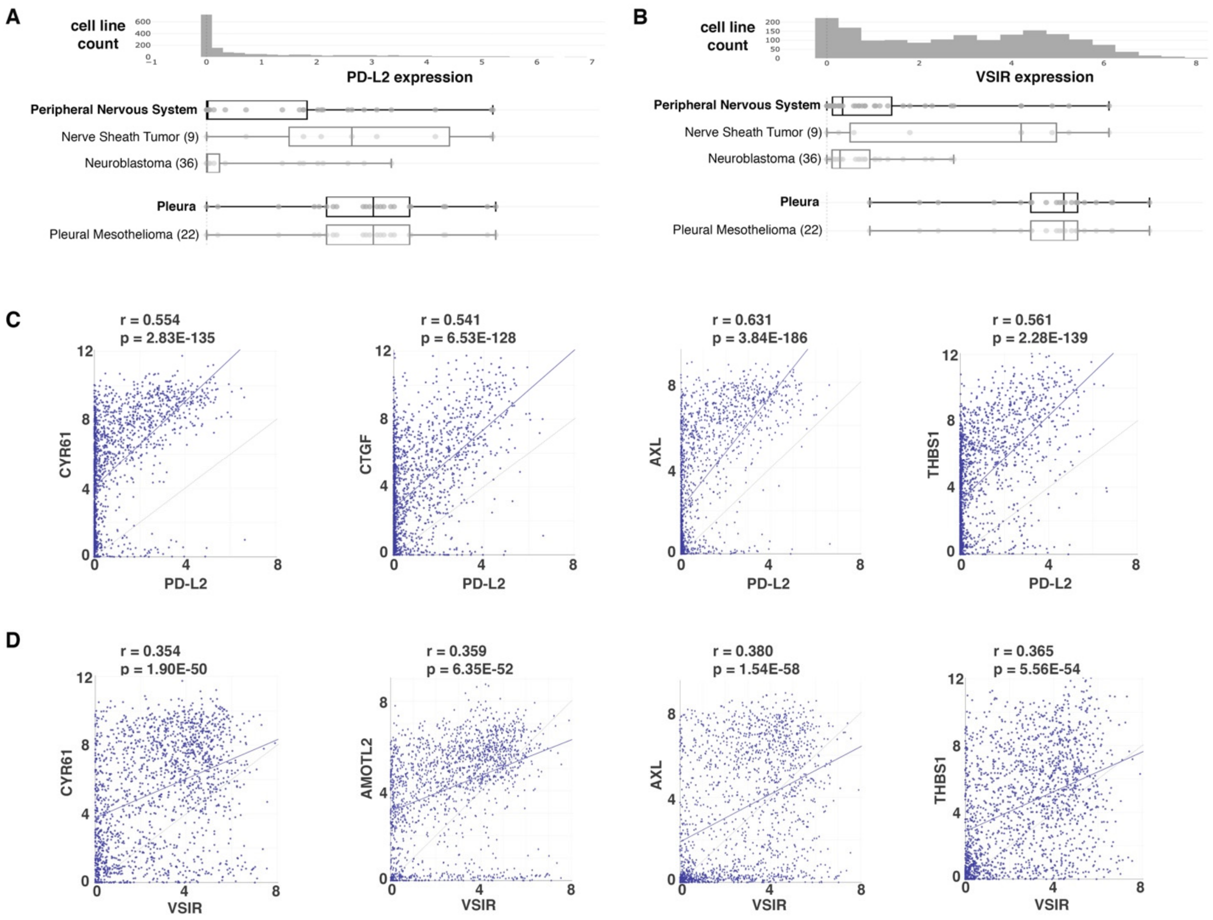

Figure S3. Expression of PD-L2 and VSIR correlates with YAP activity in human cell lines.
(**A**, **B**) DepMap expression pattern of PD-L2 (A) and VSIR (B) in indicated human cell lines. Most mesothelioma cell lines express these two genes. For comparison, few neuroblastoma cell lines express them. Each dot represents a cell line.
(**C**, **D**) Expression of PD-L2 (C) and VSIR (D) correlates with that of indicated YAP signature target genes in 1,673 human cell lines. Linear regression lines, Pearson correlation (r), and p value of linear regression are shown. Each dot represents a cell line.
